# THE ONE ANASTOMOSIS GASTRIC BYPASS TECHNIQUE: RESULTS AFTER ONE YEAR OF FOLLOW-UP

**DOI:** 10.1590/0102-672020190001e1476

**Published:** 2019-12-20

**Authors:** Gabriela RUIZ-MAR, Alondra RUELAS-AYALA, Luis Alfredo ORNELAS-OÑATE, Jorge Enrique RAMIREZ-VELASQUEZ

**Affiliations:** 1 Department of Bariatric Surgery, Hospital General de Mexico, Ciudad de México, CDMX, Mexico

**Keywords:** Obesity, Anastomosis, Gastric bypass, Obesidade, Bypass gástrico, Anastomose

## Abstract

**Background:**

Obesity is a major health problem. One anastomosis-gastric bypass (OAGB) is a restrictive and malabsorptive weight loss surgery that carries the same characteristics of Roux-en-Y gastric bypass in its status as a weight loss mechanism; but, its results remain controversial.

**Aim:**

To describe the technique and outcomes of OAGB and its effects on weight loss and remission of comorbidities.

**Methods:**

Retrospective review of all patients who underwent OAGB procedure from January 2017 to January 2018. Patients’ baseline characteristics were recorded. The routine in follow-up were at 1, 3, 6 and 12 months.

**Results:**

A total of 51 patients underwent OAGB. The mean age was 43.8±9.3 years, mean weight was 125±31 and mean BMI was 55.8±12 kg/m^2^. With regard to comorbidities, 64.7% had type 2 diabetes mellitus (T2DM), 43.1% systemic arterial hypertension (SAH) and 51% dyslipidemia. The BMI decreased for 48.4±1.3 to 31±4.4 at 12 months (p=0.0001) and we obtained an average decreased of 65% excess weight loss (EWL) at 12 months of follow-up. There was improvement in the values of total cholesterol (CT) (p=0.348); triglycerides (TGC) (p=0.0001); LDL (p=0.06), HDL (p=0.029) and A1C (p=0.405). Remission of T2DM al 12 months follow-up after surgery was 57% (p=0.124), remission of SAH 37% (p=0.040) and remission of dyslipidemia of 43% (p=0.967).

**Conclusions:**

OAGB is a commonly performed and safe procedure. Short term results appear promising; however, long-term follow-up is necessary to evaluate complications and possible nutritional effects.

## RESUMO

**Racional:**

A obesidade é um grande problema de saúde. Bypass gástrico com uma anastomose (OAGB) é operação de perda de peso restritiva e disabsortiva que carrega as mesmas características do bypass gástrico em Y-de-Roux em seu status de mecanismo de perda de peso, mas seus resultados permanecem controversos.

**Objetivo:**

Descrever a técnica e os resultados do OAGB e seus efeitos na perda de peso e na remissão de comorbidades.

**Método:**

Revisão retrospectiva de todos os pacientes que foram submetidos a OAGB de janeiro de 2017 a janeiro de 2018. As características basais dos pacientes foram registradas. O acompanhamento de rotina foi de 1, 3, 6 e 12 meses.

**Resultados:**

Um total de 51 pacientes foi submetido a OAGB-HGM. A idade média foi de 43,8±9,3 anos, o peso médio foi de 125±31 e o IMC médio de 55,8±12 kg/m^2^. Em relação às comorbidades, 64,7% apresentavam diabete melito tipo 2 (DM2), 43,1% hipertensão arterial sistêmica (HAS) e 51% dislipidemia. O IMC diminuiu para 48,4±1,3 a 31±4,4 em 12 meses (p=0,0001) e a redução média foi de 65% de perda de excesso de peso (EWL) em 12 meses de seguimento. Houve melhora nos valores do colesterol total (CT) (p=0,348); triglicéridos (TGC) (p=0,0001); LDL (p=0,06), HDL (p=0,029) e A1C (p=0,405). A remissão do DM2 ao seguimento de 12 meses após a operação foi de 57% (p=0,124), remissão da HAS 37% (p=0,040) e remissão da dislipidemia de 43% (p=0,967).

**Conclusões:**

OAGB-HGM é procedimento comumente realizado e seguro. Resultados de curto prazo parecem promissores; no entanto, o acompanhamento em longo prazo é necessário para avaliar as complicações e possíveis efeitos nutricionais

## INTRODUCTION

Obesity is a major health problem. It is known that it leads to numerous comorbidities such as cardiovascular disease, metabolic syndrome and increased mortality. Therefore, bariatric surgery was introduced as the treatment of choice for morbid obesity and has been shown to be effective in weight control and remission of comorbidities^9^. 

According to the worldwide bariatric survey the total number of bariatric/metabolic procedures performed in 2016 was 685,874. The most performed primary surgical bariatric/metabolic procedure was sleeve gastrectomy, followed by Roux-Y gastric bypass (RYGB), and in third place one anastomosis gastric bypass (OAGB)^2^.

OAGB is a restrictive and malabsorptive weight loss surgery that carries the same characteristics of Roux-en-Y gastric bypass in its status as a weight loss mechanism. Yet, it has other advantages as it is considered a simpler technique with a small learning curve and shorter operative time, and similar outcomes in terms of weight loss and remission of comorbidities^6^.

This study aims to describe the technique and outcomes of OAGB and its effects on weight loss, and on the remission of comorbidities in short follow-up. 

## METHOD

We retrospectively reviewed all patients who underwent OAGB in Hospital General de México, Ciudad de Mexico, Mexico, from January 2017 to January 2018. Patients were eligible for the OAGB if they had a body mass index (BMI) of 40 kg/m^2^ or a BMI between 35-40 kg/m^2^ with obesity related comorbidities. 

Patients’ baseline characteristics such as BMI and biochemical data were recorded. Perioperative data (operative duration, length of hospital stay) and complications were evaluated. The first postoperative follow up was done one month after the surgery. The routine follows up were scheduled at 3, 6 and 12 months. Remission of type 2 diabetes mellitus (T2DM) was defined as fasting plasma glucose levels less than 126 mg/dl in addition to HbA1c values less than 6.5% without the use of anti-glycemic therapy.

### Surgical technique

**FIGURE 1:**
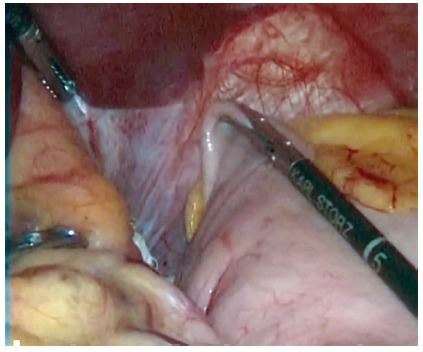
Dissection the esophagogastric (His) angle and placement of reference gauze

Surgery takes place with the patients under general anesthesia. Pneumoperitoneum is induced by the insertion of a Veress needle into the left subcostal region, until reaching an intra-abdominal pressure of 14 mmHg. The insertion of the first optic trocar (12 mm) mid-way between the xiphoid and the umbilicus, slightly left to the midline, is followed by the insertion of the rest of trocars under vision. The procedure is initiated by dissecting the esophagogastric angle (His, Figure 1). 

#### Confection of the gastric pouch

**FIGURE 2:**
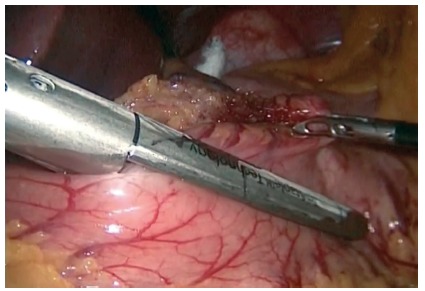
Confection of gastric pouch through the retro gastric window

Initially, identification of the lowest part of the incisura angularis of the stomach to 5 cm of pylori is done; dissection is started at the perigastric fat until reaching the lesser sac. The gastrectomy part of the procedure is started by the horizontal stapling of the stomach, using a 45-mm stapler at incisura level directed towards the greater curvature without sectioning it (Figure 2), continuing with vertical stapling high up until the esophagogastric angle. Bougie size used to create the OAGB pouch is 36Fr and a measure of the adequate length of the gastric pouch is that it protrudes from the hepatic edge. 

#### Intestinal count and restriction

The first part of jejunum at the duodenojejunal ligament (Treitz) is identified to raise the greater omentum and the transverse colon, measuring the entire small bowel length. In our technique, the length of the biliary handle (excluded) is calculated through percentages. An exclusion of 30% of total length of the intestine is performed in patients with grade II or III obesity, 35% in patients with any degree of obesity and T2DM and 40% in patients with super obesity or in revision surgeries.

#### Anastomosis

Gastrostomy is performed at the lower part of the gastric pouch, in the gastric anterior face and enterotomy in the antimesenteric portion of intestine. Gastrojejunal anastomosis is done by using a 45 mm stapler at 3.5 mm of length. The enterotomy opening is closed using PDS 3-0 in two planes. A leak test with methylene blue is performed at the end of the procedure. Finally, the removal of the trocars under control of vision and the closure of the skin with absorbable surgical thread. Patients begin ambulation and liquid diet at 2-4 h after surgery and they leave after 24 h of surgery. 

**FIGURE 3:**
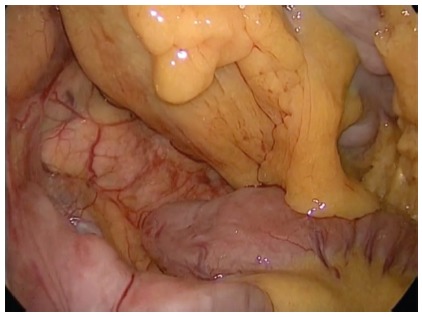
Identification of the duodenojejunal ligament (Treitz) and the entire small bowel length is measured

## RESULTS

A total of 51 patients underwent OAGB as a primary procedure (43 female, 8 male). The mean age was 43.8±9.3 years (range 19-52), mean weight was 125±31 and mean BMI was 55.8±12 kg/m^2^ (range 31-77). With regard to comorbidities, 64.7% had T2DM, 43.1% systemic arterial hypertension (SAH) and 51% dyslipidemia. All patients successfully completed the 1-year follow-up for weight loss. Patient´s baseline characteristics are presents in Table 1. 

**TABLE 1:** Patients characteristics at baseline

Characteristics		Mean±SD
Age (years)		43.8±9.3
Weight (kg)		125±31
BMI (kg/m^2^)		48.2±9.5
Gender, n (%)		
Male		8 (15.7)
Weight (kg)		171±32
BMI (kg/m^2^)		55.8±12
Female		43 (84.3)
Weight (kg)		117.2±22
BMI (kg/m^2^)		47±8.5
Co-morbidities, n (%)		
T2DM		33 (64.7)
SAH		22 (43.1)
Dyslipidemia		26 (51)

Data are presented as the mean ±SD; BMI=body mass index; T2DM=type 2 diabetes mellitus; SAH=systemic arterial hypertension

Within 30 days post-OAGB, no mortality, anastomotic leak or bleeding was reported. The BMI decreased from 48.4±1.3 to 31±4.4 at 12 months (p=0.0001) and we obtained an average decreased of 65% excess weight loss (EWL) at 12-months follow-up. There was improvement in the values of total cholesterol (CT) (for 169±42 to 154±27; p=0.348); triglycerides (TGC - for 151±74 to 97±58; p=0.0001); LDL (for 106±29.2 to 87±21; p=0.06), HDL (for 39.8±8.6 to 48±9.9; p=0.029) and A1C (for 6.03±1.5 to 5.56±0.7; p=0.405, Table 2). Remission of T2DM al 12 months follow-up after surgery was 57% (p=0.124), remission of SAH 37% (p=0.040) and remission of dyslipidemia of 43% (p=0.967). 

**TABLE 2:** Postoperative follow-up in patients after OAGB

	Pre-operative	Post-operative 12 months	p
Weight	125± 31	80±14.4	0.0001
BMI	48.4± 1.3	31±4.4	0.0001
A1C	6.03±1.5	5.56±0.7	0.405
CT	169±42	154±27	0.348
TGC	151±74	97±58	0.0001
LDL	106±29.2	87±21	0.06
HDL	39.8±8.6	48±9.9	0.029

Data are presented as the mean±SD; BMI=body mass index; CT=total cholesterol; TGC=triglycerides; LDL, LDL-cholesterol; HDL=HDL-cholesterol; A1C=glycosylated hemoglobin

Gastrointestinal endoscopy was performed on all patients. Prior to the surgical procedure, nine (17.6%) presented some degree of esophagitis; four (7.4%) esophagitis A and five (9.8%) esophagitis B. In the control endoscopy 12 months after the procedure, esophagitis de novo was not reported; normal esophagus was reported in 100% of patients who presented esophagitis A in the preoperative period; among those with grade B esophagitis, normal esophagus was reported in four and one progressed to esophagitis C. Two (3.9%) had biliary reflux and other two (3.9%) gastrojejunal anastomotic ulcers in postoperative period. 

## DISCUSSION

The one anastomosis gastric bypass has been presented as an option of surgical treatment for obese patients in order to reduce operation time and avoiding eventual postoperative complications. Has been reported that the results with this procedure in terms of weight loss, BMI reduction and improvement of comorbidities are quite similar to the RYGB and sleeve gastrectomy. However, a potential risk of complications related to bile reflux is possible, even if modifications of the technique, in order to prevent it, have been introduced^3^. As described for the simplified RGYB technique^1^, the OAGB has been associated with a decrease in surgical time, postoperative complications and the learning curve necessary for its realization.

In the study of IFSO Middle East North Africa 2019, bougie size used to create the OAGB pouch varied from 32 to 40 Fr with 36 Fr bougie being most commonly used in 67%^5^. In our group the results have been obtained with bougie of 36 Fr without stenosis or dysphagia symptoms. 

Although the use of drainage has been reported in up to 44% of the groups, we have not found complications without using it. Has been reported that only 28% of surgeons measured the entire small bowel length^5^; however, we believe it is important to know it to avoid long-term nutritional complications. Carbajo^4^ reported 70% EBWL at 12-year follow-up in his patient population where he bypassed 50% of the total length of small bowel. In the present study, with the percentage exclusion of the small intestine, we obtained an average decreased of 65% EBWL at 12 months follow up. A systematic review of OAGB studies revealed that the mean EWL% at 12 months ranged between 55-88%^6^.

The present study has shown metabolic improvement following OAGB among T2DM patients with remission rate of 57% after 12 months follow-up. This percentage is lower than the reported in other studies from 80-87.5%^6^ ^,^ ^7^ ^,^ ^8^. These results may be related to the fact that in our population the diabetes rates and their complications are among the largest reported worldwide; regarding populations, the results in diagnosis and intervention in diabetes are made earlier; so, time may be different.

Bile reflux has been one of the main discussed complications of the OAGB. In a study where it was done, an analysis of the reported experiences with Billroth II gastrectomy technique, reported high gastrointestinal symptoms, Barrett esophagus and esophageal carcinoma^3^. However, the surgical technique in Billroth II anastomoses and in OAGB are different and with different indications, therefore the results cannot be comparable. The incidence of bile reflux after OAGB has been reported in the range of 0.4-4%^6^ and the marginal ulcer rate has been reported 0.2-1.7%^8^. In our institution, we do preoperative endoscopy in all patients independently of their symptoms. We reported 1.9% of esophagitis progression and 88.8% remission after surgical procedure. These results haven’t been reported previously because endoscopy is not performed as part of the international protocol.

The initial experience of our center is presented; however, long-term follow-up is necessary to evaluate the development of nutritional complications, the presence of bile reflux and the quality of life of patients. 

## CONCLUSION

OAGB is a commonly performed and safe procedure. Short term results appear promising; however, long-term follow-up is necessary to evaluate complications and possible nutritional effects. 
